# The Prediction of Preterm Birth Using Time-Series Technology-Based Machine Learning: Retrospective Cohort Study

**DOI:** 10.2196/33835

**Published:** 2022-06-13

**Authors:** Yichao Zhang, Sha Lu, Yina Wu, Wensheng Hu, Zhenming Yuan

**Affiliations:** 1 Hangzhou Normal University Hangzhou China; 2 Department of Obstetrics and Gynecology Hangzhou Women's Hospital Hangzhou China; 3 Department of Obstetrics and Gynecology The Affiliated Hangzhou Women’s Hospital of Hangzhou Normal University Hangzhou China

**Keywords:** preterm birth prediction, temporal data mining, electronic medical records, pregnant healthcare

## Abstract

**Background:**

Globally, the preterm birth rate has tended to increase over time. Ultrasonography cervical-length assessment is considered to be the most effective screening method for preterm birth, but routine, universal cervical-length screening remains controversial because of its cost.

**Objective:**

We used obstetric data to analyze and assess the risk of preterm birth. A machine learning model based on time-series technology was used to analyze regular, repeated obstetric examination records during pregnancy to improve the performance of the preterm birth screening model.

**Methods:**

This study attempts to use continuous electronic medical record (EMR) data from pregnant women to construct a preterm birth prediction classifier based on long short-term memory (LSTM) networks. Clinical data were collected from 5187 pregnant Chinese women who gave birth with natural vaginal delivery. The data included more than 25,000 obstetric EMRs from the early trimester to 28 weeks of gestation. The area under the curve (AUC), accuracy, sensitivity, and specificity were used to assess the performance of the prediction model.

**Results:**

Compared with a traditional cross-sectional study, the LSTM model in this time-series study had better overall prediction ability and a lower misdiagnosis rate at the same detection rate. Accuracy was 0.739, sensitivity was 0.407, specificity was 0.982, and the AUC was 0.651. Important-feature identification indicated that blood pressure, blood glucose, lipids, uric acid, and other metabolic factors were important factors related to preterm birth.

**Conclusions:**

The results of this study will be helpful to the formulation of guidelines for the prevention and treatment of preterm birth, and will help clinicians make correct decisions during obstetric examinations. The time-series model has advantages for preterm birth prediction.

## Introduction

### Background

Preterm birth, defined as birth occurring before 37 weeks of completed gestation, is the primary cause of neonatal death and disability and affects the long-term health of newborns [1,2]. According to the World Health Organization global action report on preterm birth, there are approximately 15 million premature infants born in the world every year, with an incidence rate of 5% to 18%; 1 million of these premature infants die [3]. China is the most populous country in the world, and the implementation of the two-child policy has increased the average age of first pregnancy and the incidence of preterm birth [4-6]. Compared to full-term birth, prematurity imposes adverse effects on the health and safety of both the pregnant woman and the infant. Prematurity increases the incidence of congenital malformation, being small for gestational age, and nervous system diseases associated with immature organs [7-9]. Therefore, early prediction of preterm birth and preventive measures have a significant potential to reduce mortality and improve the survival rate of preterm infants [10,11].

Despite the serious clinical consequences, there are currently no effective early screening methods for preterm birth. It is generally considered that ultrasonography cervical-length assessment is the most effective screening method [11,12], but routine, universal cervical-length screening remains controversial because of its cost [13,14]. Cervical screening is not popular in China and is performed only for pregnant women with cervical insufficiency [15]. Fetal fibronectin is an extracellular matrix glycoprotein that has also been extensively studied as a predictor of preterm birth, and although it has high specificity, it has a low detection rate [16]. Other biomarkers, including inflammatory factors, serum proteomics, and genetic factors, are associated with preterm birth [17], but each of these only has good performance in a subset of cases, and few studies have demonstrated that they are sufficiently useful for clinical use.

There is not a single or combined screening method for preterm birth that has high sensitivity and can reliably identify women at risk for preterm birth [11]. The etiological mechanism of preterm birth is elusive, and the interaction between risk factors is complex. Machine learning algorithms based on time-series technology can solve nonlinear relationships between multi-dimensional variables and analyze and mine their time-series characteristics. These machine learning models have been shown to be effective in the prediction of obstetric diseases [18,19]. Therefore, this paper proposes a time-series preterm birth prediction model based on a long short-term memory (LSTM) network.

### Related Work

In the literature, various methods have been proposed to predict the risk of preterm birth with machine learning. These methods can be broadly categorized into 2 types, according to their data source: special examination data or routine clinical data. Special examination data include findings from the cervicovaginal fluid [20], electrohysterography [21], and whole-blood gene expression [22]. These data need special methods to obtain and are not suitable for large-scale initial screening. Therefore, research results based on these data have only been shown to have better prediction performance in small-sample data sets. Other research has sought to build prediction models based on routine clinical examination data and demographic data. Koivu et al [23] used a US Centers for Disease Control and Prevention (CDC) data set of almost sixteen million observations to build a prediction model; the best-performing machine learning model achieved an area under the curve (AUC) of 0.64 for preterm birth when using external the New York City test data. Lee et al [24] used the same CDC and New York City data sets to build an artificial neural network prediction model; it also had an AUC of 0.64. Weber et al [25] assessed the prediction of early (<32 weeks) spontaneous preterm birth among non-Hispanic women by applying machine learning to multilevel data from a large birth cohort; the AUC of this prediction model was 0.67.

Although the above prediction models have relatively reliable performance, they all use huge, complex data sets for analysis. It can be difficult to obtain complete data sets of this size and complexity because of privacy issues. More importantly, these models ignore the influence of time-related factors. Time-series analysis and prediction methods predict future developments according to tendencies in past changes and highlight the role of time factors in making predictions. In fact, obstetric examinations are continuous and repeated time-series records and are considered to be related to pregnancy risk [26]. Previous studies have reported that time-series models perform well in the field of obstetrics. For example, Tao et al [27] used maternal weight change trajectories during pregnancy to establish a time-series hybrid model to predict the birth weight of newborns. Zhou et al [28] predicted the risk of postpartum hemorrhage using continuous data from prenatal physical examinations. Compared with other biological phenomena, the 280-day gestational cycle has a relatively fixed time; pregnant women also have high compliance to obstetric outpatient examinations [29]. Therefore, a time-series model to mine time-series characteristics from data obtained during pregnancy has high potential.

Few studies have described the interpretability of their models. Khatibi et al [30] used Iran’s national databank of maternal and neonatal records to design a map/reduce phase-based, parallel feature selection machine learning algorithm to predict the risk of preterm birth. The map phase used parallel feature selection and classification methods to score features, while the reduce phase aggregated the feature scores in order to determine the contribution of predictors to the model. Similar methods include the calculation of frequency statistics, the Gini index and other indicators that trace the decision-making process of the tree model [31], and calculating Shapley values to define the importance of features [32].

Although none of the above methods are suitable for time-series models, it is encouraging that there have been recent proposals for interpretable frameworks for time-series classification that can be used in different medical scenarios. In the field of medical signals, Ivatur et al [33] proposed a post-hoc explainability framework for deep learning models applied to quasi-periodic biomedical time-series classification that included 3 different techniques for explanation: studying ablation, studying permutation, and using a local, interpretable model-agnostic explanation method. Maweu et al [34] proposed a modular framework named the convolutional neural network (CNN) explainability framework for electrocardiogram signals that explains the quality of the deep learning model in terms of quantifiable metrics and feature visualization. Electronic medical record (EMRs) contain time series and multimodal data which further hinder interpretability. Nguyen-Du et al [35] proposed a new deep electronic health record spotlight framework for transforming EMR data into pathways and 2D pathway images, which can then be used with 2D CNN techniques to support visual interpretation. Viton et al [36] proposed an approach based on heat maps as a visual means of highlighting significant variables over a temporal sequence, which can be applied to the problem of predicting the risk of in-hospital mortality.

This previous research motivated the current study, which makes the following key contributions: (1) we designed and implemented a complete process for preterm birth screening and providing early warnings based on regular EMR data; (2) we used machine learning based on time-series technology to analyze the obstetric examination data and improve the performance of the prediction model; (3) we provide a preliminary explanation of the quantitative interpretability of the model and explore time-series predictors affecting preterm birth.

## Methods

### Setting and Study Population

**Figure 1:**
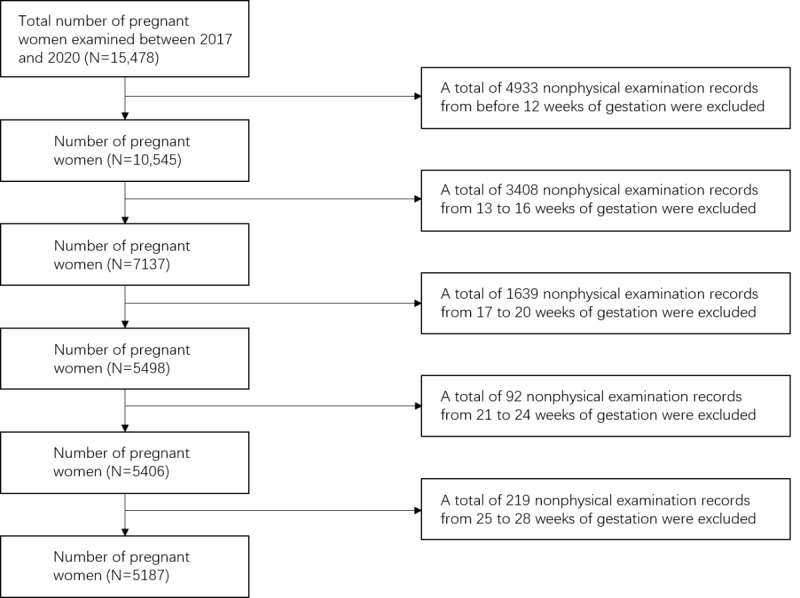
Flow chart showing participant selection.

The data were collected from Hangzhou Women’s Hospital (Hangzhou Maternity and Child Health Care Hospital), Hangzhou, Zhejiang Province, China, between 2017 and 2020. This study included >25,000 pregnant women who received antenatal care at Hangzhou Women’s Hospital and eventually gave birth naturally through the vagina. The exclusion criteria were as follows: presence of multiple pregnancies, assisted reproduction, severe cardio- or cerebrovascular complications or comorbidities, and performance of cervical cerclage during pregnancy. The inclusion criterion was a first pregnancy test taken before 12 gestational weeks. According to the Chinese guidelines for prenatal examination [37], pregnant women should have a monthly outpatient examination before 28 weeks of gestation. Figure 1 shows the filtering and processing flow chart used to select the study population. Some women were excluded owing to failure to obtain data or implausible pregnancy outcomes. Data from a final total of 5187 women were available for analysis.

### Clinical Measurements and Data Collection

Demographic data, physical examination data, ultrasound records, and laboratory data from the antenatal period were retrieved from EMRs. At registration for pregnancy, information on maternal demographic characteristics (eg, age, education, and occupation), anthropometrics (eg, body weight, height, and blood pressure), and clinical history (eg, parity and disease history) were recorded. As shown in Table 1, repeated pregnancy data were obtained for each individual from the first pregnancy test to the final pregnancy test, taken between 25 to 28 weeks. The clinical data included age, weight, uterine height, abdominal circumference, blood pressure, and findings from ultrasonic examination. Laboratory tests (eg, routine blood examination and blood biochemistry examination, including blood lipids and glucose) were performed at 24 weeks of gestation.

Participants were asked to wear light clothing when their height and weight were measured. BMI was calculated as body weight in kilograms divided by body height in meters squared. Sitting blood pressure was examined after at least 10 minutes of rest using a standard mercury sphygmomanometer with the patient’s right arm held at heart level. Maternal venous blood samples were drawn in the morning after an overnight fast of ≥8 hours.

**Table 1:** Description of data sources.

Gestational age	Ultrasonic examination	Laboratory tests
Before 12 weeks	✓^a^	N/A^b^
From 13 to 16 weeks	✓	N/A
From 17 to 20 weeks	✓	N/A
From 21 to 24 weeks	✓	N/A
From 25 to 28 weeks	✓	✓

^a^✓ indicates that the pregnant woman has made relevant clinical examination in this pregnancy stage.

^b^N/A: not applicable.

### Model Design

Based on the above-mentioned features, 2 machine learning models were constructed to predict preterm birth. One was an early prediction model based on the data sources in Table 1. For each cross-sectional gestational age category, extreme gradient boosting (XGB) combined with decision trees was employed to establish the prediction model. XGB is an improvement on the gradient lifting algorithm and is widely used in the field of obstetric auxiliary diagnosis [38]. The second model used temporal prediction techniques. Long short-term memory networks (LSTMs) are a type of time-cyclic neural network that are suitable for processing and predicting events with relatively long intervals and delays in the time series [39]. LSTMs can avoid the gradient disappearance of conventional recurrent neural networks and are widely used in the field of disease diagnosis [40].

LSTMs realize information protection and control through 3 control gates, namely the input gate, the forgetting gate, and the output gate. The key in LSTMs is the unit state. The LSTM unit judges whether the output of the previous time step is useful; only useful information is saved and the rest is forgotten at the forgetting gate. Equations (1) through (5) represent the parameter update process, where σ represents the sigmoid function, h_t–1_ represents the output of the LSTM at the previous time step, and h_t_ represents the current output; *I*, *f*, and *o*, respectively, represent the input gate, forgetting gate, and output gate in the LSTM unit. Equation (4) represents the process of the state transition of the memory unit, where c_t_ is the state of the memory unit at the current time step. The current state is calculated by the previous time step state, c_t–1_, and the result of the forgetting gate and the input gate of the current-time LSTM unit.

i_t_ = σ (W_χi_χ_t_ + W_hi_h_t-1_ + b_i_) **(1)**

f_t_ = σ (W_χf_χ_t_ + W_hf_h_t-1_ + b_f_) **(2)**

o_t_ = σ (W_Xo_χ_t_ + W_ho_h_t-1_ + b_o_) **(3)**

C_t_ = f_t_c_t-1_ + i_t_tanh(W_xc_χ_t_ + W_hc_h_t-1_ + b_c_) **(4)**

h_t_ = o_t_tanh(c_t_) **(5)**

The parameters of these prediction models were determined by grid search. The models were validated with 5-fold cross-validation. The 5-fold cross-validation splits the training dataset into 2 sections, where 80% of the dataset is used for training and the remaining 20% is used for testing. Simultaneously, the incidence rate of preterm birth is about 5%, so in situations where there were imbalanced class data combined with unequal error costs, random oversampling was used to balance the dataset to get true performance values for the classifier. The random oversampling method makes the number of minority classes the same as the number of majority classes by randomly copying minority class samples to get new equilibrium data.

Under the Python 3.6 environment (Python Software Foundation), the data analysis and visualization were completed by using NumPy, Pandas, Matplotlib, Seaborn, and other libraries [41,42]. The machine learning model comes from the scikit-learn library and the deep learning framework adopts PyTorch [43]. Based on the amount of data in this study, the LSTM network was able to run on a personal computer. The adaptive learning rate of the Adam optimizer [44] was used to accelerate the convergence speed of the LSTM model. Table 2 shows the values of the parameters for the 2 models.

**Table 2:** Summary of parameter values in each model.

Parameters		Values
**Extreme gradient boosting model**		
	Learning rate	0.01
	N_estimators	200
	Min_samples_leaf	4
	Min_samples_split	3
	Max_depth	2
**Long short-term memory model**		
	Loss function	CrossEntropy
	Num_layers	2
	Optimizer	Adam
	Hidden_size	130
	Input size	65
	Learning rate	0.001
	Batch-size	256
	Epochs	20

### Model Evaluation

The characteristics were compared between the preterm birth and full-term birth groups. Statistical tests were 2-sided; *P* values <.05 were considered statistically significant. All analyses were performed using the statistical software SPSS 22.0 (IBM).

The prediction performance was considered an important factor to evaluate the proposed model. In this paper, the receiver operating characteristic (ROC) curve and AUC were used to evaluate the model’s ability to predict preterm birth. In addition, the evaluation indicators of the confusion matrix, including accuracy, sensitivity, and specificity, were used to analyze the relationship between the actual values and the predicted values for the risk of preterm birth. Accuracy, sensitivity, and specificity were calculated as follows: accuracy = (TN + TP) / (TN + TP + FN + FP); sensitivity = TP / (TP + FN); and specificity = TN / (TN + FP), where TP indicates true positive, FP indicates false positive, TN indicates true negative, and FN indicates false negative.

Feature importance reflects the contribution each variable makes in classifying preterm birth, which explains the results of the model decision. In this study, feature importance for the XGB model was calculated by the sum of the decrease in error when split by a variable [31]. For the LSTM model, feature ablation was used, which provides feature importance at a given time step for each input feature [45], computing attribution as the difference in output after replacing each feature with a baseline; a lower AUC indicates a more important feature.

### Ethics Approval

The study design was approved by the local Ethical and Research Committee (written permission, with approval number 2019-02-2). All medical procedures were performed following the relevant guidelines and regulations. The informed consent requirement for this study was waived by the board because the researchers only accessed the database for analysis purposes and all patient data were deidentified. 

## Results

### General Characteristics of the Study Participants

The data set used in this paper comes from a hospital in eastern China and is very extensive, including maternal ultrasound records, prenatal examination reports, and laboratory data. Of the 5187 pregnant women enrolled in the present study, 4966 gave birth at full term. The remaining 221 women gave birth preterm. The general characteristics of the participants are presented in Table 3. Table 4 summarizes the clinical characteristics of the study subjects at the second trimester (25-28 weeks).

**Table 3:** General characteristics of the study population (N=5187)

Characteristics	Mean (SD)
Age, years	29.63 (3.52)
Prepregnancy weight, kg	53.65 (8.15)
Height, cm	161.45 (4.84)
Prepregnancy BMI, kg/m^2^	20.57 (2.92)
Parity, number	0.26 (0.46)
Gravidity, number	1.71 (0.98)
Prepregnancy SBP^a^, mmHg	106.12 (13.02)
Prepregnancy DBP^b^, mmHg	67.29 (9.31)
Number of preterm births in reproductive history, parity number	0.003 (0.05)
Menarche, years	13.47 (1.22)
Period, days	6.07 (3.03)
Cycle, days	29.55 (7.06)

^a^Systolic blood pressure.

^b^Diastolic blood pressure.

**Table 4:** Clinical characteristics and laboratory parameters at the second trimester.

Characteristics		Full-term birth (n=4966)	Preterm birth (n=221)	*P* value
		Mean (SD)	Mean (SD)	
**General characteristics**				
	Age, years	29.61 (3.49)	30.14 (3.64)	.02
	Prepregnancy weight, kg	53.92 (7.16)	53.74 (8.12)	.31
	Prepregnancy SBP^a^, mmHg	106.70 (10.45)	106.19 (11.98)	.48
	Prepregnancy DBP^b^, mmHg	67.65 (7.96)	67.47 (7.41)	.53
**Physical data**				
	Gestational age, weeks	26.02 (1.17)	26.09 (1.19)	.73
	Pulse rate, beats per minute	77.63 (7.27)	77.32 (6.82)	.56
	Maternal weight at pregnancy, kg	61.16 (7.28)	60.39 (8.29)	.29
	SBP, mmHg	111.42 (10.62)	113.19 (11.24)	.04
	DBP, mmHg	65.29 (7.78)	66.09 (8.20)	<.001
	Uterine height, cm	24.48 (1.82)	24.02 (2.28)	.45
	Mother abdominal circumference, cm	88.76 (5.45)	86.98 (8.33)	.45
**Ultrasonic data**				
	Biparietal diameter, cm	6.70 (0.23)	6.84 (0.48)	.05
	Head circumference, cm	24.60 (0.76)	25.02 (1.47)	.13
	Femur length, cm	4.83 (0.17)	4.93 (0.34)	.06
	Fetal abdominal circumference, cm	22.18 (0.86)	22.94 (1.45)	.03
**Laboratory data**				
	Triglyceride, mmol/L	2.15 (0.78)	2.25 (0.79)	.02
	Total bile acid, µmol/L	2.22 (1.75)	2.17 (1.52)	.43
	Uric acid, µmol/L	244.05 (49.69)	246.05 (49.60)	.12
	Platelets, cells × 10^9^/L	209.12 (45.24)	212.26 (46.10)	.11
	Fasting blood glucose, mmol/L	4.35 (0.38)	4.40 (0.46)	.04
	Total cholesterol, mmol/L	6.23 (1.01)	6.19 (1.07)	.28
	Activated partial thromboplastin time, seconds	26.25 (2.97)	26.26 (3.31)	.75
	Fibrinogen, g/L	3.77 (0.63)	3.85 (0.64)	.03
	Hemoglobin, g/L	115.96 (8.44)	116.79 (8.61)	.04

^a^Systolic blood pressure.

^b^Diastolic blood pressure.

### Model Performance

Based on the above-mentioned features in Table 3 and Table 4, 2 machine learning models were constructed to predict preterm birth. An XGB model was used for cross-sectional research and an LSTM model was used for time-series research. The optimal parameters were set for each predictive model and corroborated via a test data set that was derived from the training data set by 5-fold cross-validation. The accuracy, sensitivity, specificity, and AUC of the models for predicting preterm birth are shown in Table 5, which compares the performance of these 2 models in identical testing data sets. Notably, the LSTM model, used for time-series research, had the best overall prediction ability. Its accuracy, sensitivity, specificity, and AUC were 0.739, 0.407, 0.982, and 0.651, respectively. Furthermore, the model performance gradually improved with the number of gestational weeks. The overall performance of the model was best in the last cross-sectional gestational age group, with an overall accuracy of 0.689, sensitivity of 0.407, specificity of 0.979, and AUC of 0.601.

**Figure 2:**
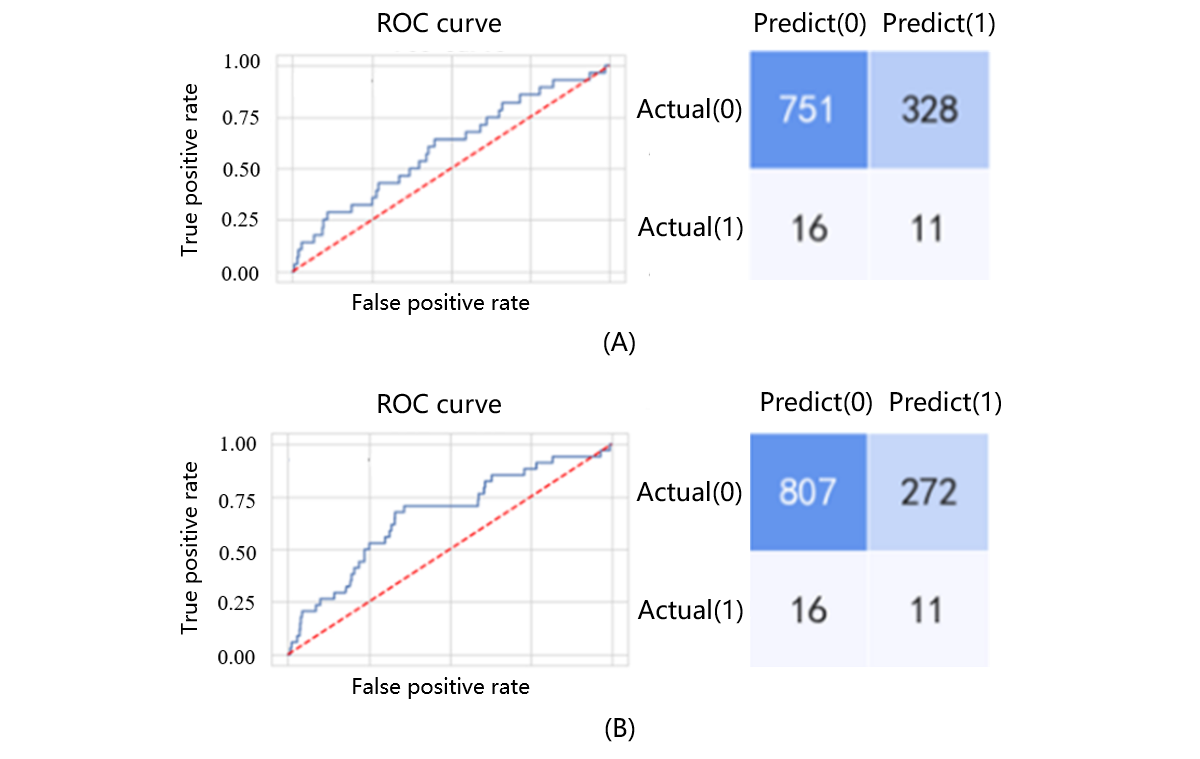
Receiver operating characteristic curves and confusion matrix of prediction models: (A) cross-sectional prediction of the extreme gradient boosting model at weeks 25 to 28; (B) prediction results of the long short-term memory model. ROC: receiver operating characteristic.

Based on the validation result for the training data set, an independent testing data set was used for predicting preterm birth. The matrices and ROC curves for the predictive models in the testing data set are shown in Figure 2. Compared with cross-sectional designs, the LSTM model had a lower misdiagnosis rate at the same detection rate. The high specificity of the model excluded more true negative samples, lowering the cost of screening.

**Table 5:** Average prediction results of different methods after 5-fold cross-validation.

Prediction results	Observation period (gestational weeks)						Time series
	Before 12 weeks	Weeks 13-16	Weeks 17-20	Weeks 21-24	Weeks 25-28		
AUC^a^	0.532	0.558	0.516	0.568	0.601	0.651	
Sensitivity	0.286	0.365	0.362	0.387	0.407	0.407	
Specificity	0.974	0.978	0.977	0.977	0.979	0.982	
Accuracy	0.525	0.574	0.584	0.622	0.689	0.739	

^a^AUC: area under the receiver operating characteristic curve.

### Influence of Variables on Predictions

**Figure 3:**
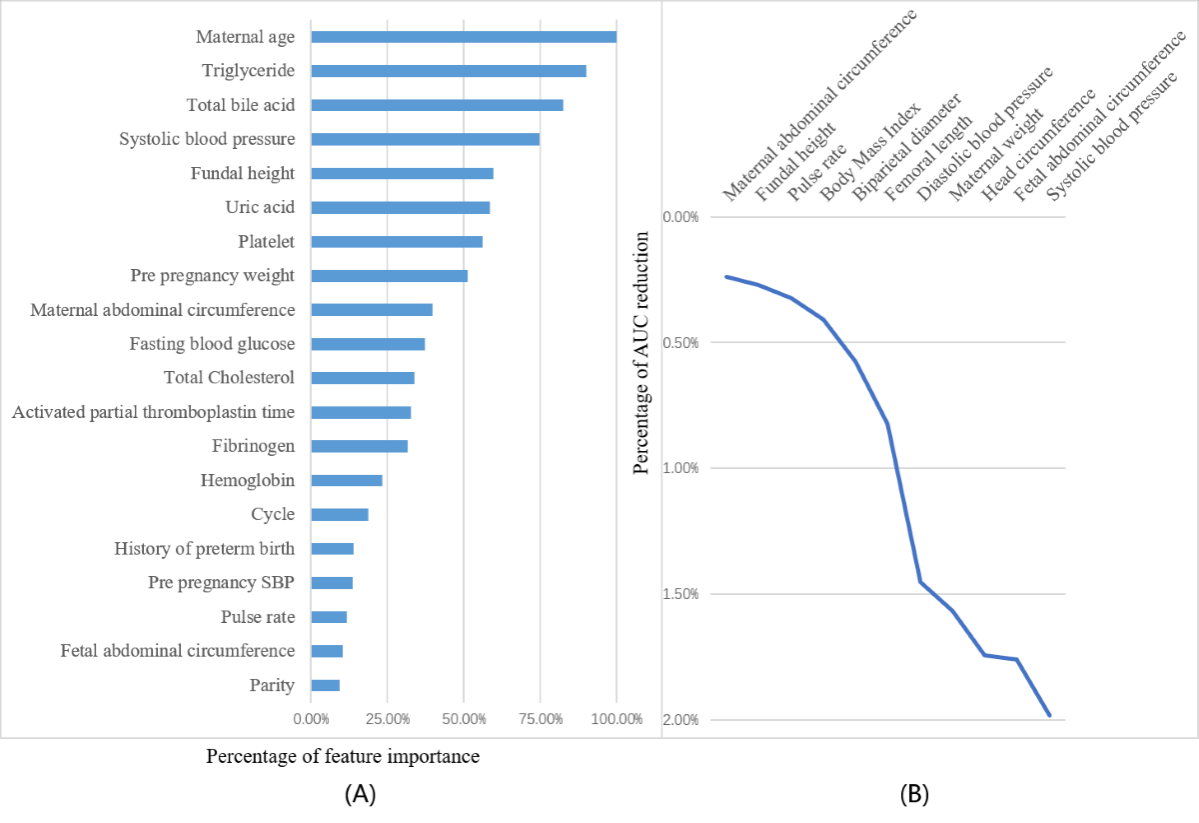
Importance of the variables: (A) identification of important features by the extreme gradient boosting model at weeks 25 to 28; (B) identification of important features by the long short-term memory model. AUC: area under the curve; SBP: systolic blood pressure.

The identification of important features by the XGB and LSTM models is shown in Figure 3. Feature importance was calculated by XGB as the sum of the decrease in error when split by a variable, which reflects the contribution each variable makes in classifying. Maternal age was the most important variable to predict preterm birth, followed by triglyceride level, total bile acid level, systolic pressure during pregnancy, fundal height, uric acid level, platelet level, and prepregnancy weight. The LSTM model for time-series research achieved the best performance, and feature ablation provided feature importance for a given time-series input feature. The importance of features was evaluated according to the degree of AUC decrease. The results indicated that the AUC decrease rate for systolic blood pressure was 2%, which was the most important time-series feature, followed by fetal abdominal circumference, head circumference, and maternal weight.

## Discussion

### Principal Findings

Premature birth is widely recognized as an increasingly serious problem. In this study, 5 pregnancy test records in the first and second trimesters of pregnancy were selected to construct a time-series model to predict preterm delivery. Compared with traditional machine learning models, the use of a time-series model improved prediction performance for preterm birth and allowed the identification of important variables for predicting preterm birth.

The early prediction of preterm birth has always been challenging. The input index of traditional prediction model research has usually been a special test item or a combination of tests that aim to find new markers that have a high contribution to preterm birth prediction; most past studies have not been clinically verified [11,17,46]. Many studies have tried to effectively predict preterm birth, which would allow early detection and prompt management. Cervical screening, fetal fibronectin measurement, or the combination of these methods can effectively predict preterm birth [12-14,16,47]. However, there are still flaws in the forecasts. For asymptomatic women, the performance of the fetal fibronectin test is too low to be clinically relevant [48]. Many studies have found that cervical status is an independent risk factor for preterm birth. In China's 2014 edition of the Clinical Diagnosis and Treatment Guidelines for Preterm Delivery [49], it is recommended that when cervical length is <25 mm, transvaginal ultrasound should be performed before 24 weeks to predict preterm birth in high-risk patients. In fact, cervical examinations are still controversial for screening of the general population. Some studies advocate for dynamic cervical examination regardless of whether a subject is high- or low-risk [50,51]. On the other hand, a greater number of studies either oppose or do not recommend large-scale cervical screening, for reasons that include but are not limited to the material cost, the time required, the lack of unified standards, and the professional training of laboratory personnel [13,14,52-55], which may lead to costs that do not conform to health economics. The prediction model in this study effectively predicts the early development of preterm labor based on demographic factors and prenatal laboratory data. These data are easy to obtain in routine clinical practice. Therefore, the prediction model of preterm birth proposed in this study can be used as a practical screening method for preterm birth in the first and second trimesters of pregnancy.

In fact, earlier works have already reported very close or even higher accuracy than this study. Compared with the large national databases used in previous studies, the conventional data used in this paper is still relatively weak, especially in its lack of key information, such as obstetric and gynecological history and family history. However, we are excited that this paper significantly improves the performance of prediction models through a machine learning method based on time-series technology.

This study reveals various new factors that affect the prediction of preterm birth. Additionally, parameters that have been traditionally reported to be related to delivery date, such as age, prepregnancy weight, history of preterm birth, and menstrual cycle, were confirmed to be influential factors in preterm birth prediction [1,56]. Interestingly, blood pressure, blood glucose, lipids, uric acid, and other metabolic factors were also very important factors related to preterm birth. Although it has not been thoroughly investigated, the relationship between metabolic risk factors and preterm birth has been preliminarily recognized in several previous studies [57,58]. In a recent observational study of 5535 deliveries, pregnant women with a cluster of metabolic risk factors during early pregnancy were more likely to give birth preterm [59]. The metabolic reaction during pregnancy normally meets the needs of fetal growth; however, an excessive metabolic stress reaction can lead to the occurrence of various pathologies in pregnancy [60]. Despite the controversy, changes in metabolic levels during pregnancy have been observed in women who give birth preterm.

### Limitations

This study has several limitations. First, the laboratory examinations of the pregnant women were completed in their respective communities before 20 weeks of gestation, precluding them from being included in the analysis due to differences in test standards. In addition, the prepregnancy characteristics were affected by recall bias; moreover, most of the included women were primipara. Thus, the contribution of preterm birth history to the model was limited. Second, the performance of the model still needs to be improved, although LSTM has great potential. Nonetheless, considering this prediction model is a baseline model based on conventional data, it can continue to add biochemical and biophysical markers to increase screening performance. In addition, advanced maternal age was a clear confounding factor [61], and stratified analysis by age will be considered in a follow-up study. Third, this paper is only a preliminary explanation of the interpretability of the machine learning model. Future work will consider using a more sophisticated post hoc explainability framework, especially for time-series problems. Finally, the study was possibly affected by selection bias due to its single-center design. The prediction model has not been widely used in clinical practice, and its accuracy and practicality should be verified in prospective studies with larger samples.

### Conclusions

Preterm birth is the primary cause of neonatal death and disability, and early prediction of preterm birth has great potential to improve the survival rate of preterm infants. In this work, we analyzed obstetric medical data based on time-series machine learning and evaluated the risk of preterm birth. Our study can screen high-risk groups for preterm birth in the early and middle trimesters of pregnancy. Compared with a traditional cross-sectional study, the time-series LSTM model in this study had better overall prediction ability with a lower misdiagnosis rate and the same detection rate. In future work, we will further improve the data set, especially regarding some key characteristics of premature birth that have been reported by past relevant research, and build a more sophisticated post hoc explainability framework for the time series model.

## Abbreviations

AUC: area under the curve
CDC: US Centers for Disease Control and Prevention
CNN: convolutional neural network
EMR: electronic medical records
FN: false negative
FP: false positive
LSTM: long short-term memory
RNN: recurrent neural network
ROC: receiver operating characteristic
TN: true negative
TP: true positive
XGB: extreme gradient boosting
